# Evaluation of the APRI score and blood-count-derived inflammatory markers in patients with preeclampsia

**DOI:** 10.1186/s12884-026-09062-9

**Published:** 2026-04-18

**Authors:** Büşra Öztürk Taş, Cenk Soysal, İsmail Bıyık, Özlem Ulaş, Yasemin Taşçı

**Affiliations:** Department of Obstetrics and Gynecology, Kutahya Health Sciences University, Kutahya, Turkey

**Keywords:** APRI score, Inflammatory markers, Preeclampsia, ROC analysis, Systemic inflammation

## Abstract

**Objective:**

This study aimed to evaluate the clinical significance of the Aspartate Aminotransferase to Platelet Ratio Index (APRI) score and blood-count-derived inflammatory markers in patients with preeclampsia.

**Materials and methods:**

This retrospective study included pregnant women diagnosed with preeclampsia, gestational hypertension, and healthy controls. Demographic, perinatal, hematological, and biochemical parameters were analyzed. Inflammatory markers, including Neutrophil-to-Lymphocyte Ratio (NLR), Monocyte-to-Lymphocyte Ratio (MLR), Platelet-to-Lymphocyte Ratio (PLR), Systemic Immune-Inflammation Index (SII), and APRI score, were compared among the study groups. ROC analysis was performed to assess the diagnostic discriminatory performance of these markers in diagnosing preeclampsia.

**Results:**

The APRI score was significantly higher in the preeclampsia group compared to the control and gestational hypertension groups (*p* = 0.004), suggesting potential hepatic involvement in preeclampsia. In contrast, no statistically significant differences were found among the groups for NLR (*p* = 0.106), MLR (*p* = 0.593), PLR (*p* = 0.319), and SII (*p* = 0.247). Hematological and biochemical analysis revealed that AST, BUN, and creatinine levels were significantly elevated in the preeclampsia group (*p* < 0.001), consistent with hepatic and renal involvement. However, platelet counts and hemoglobin levels did not show significant differences among the groups. ROC analysis demonstrated that the APRI score had poor diagnostic discriminatory performance (AUC = 0.437, *p* = 0.128), and similarly, other inflammatory markers, including NLR (AUC = 0.539), MLR (AUC = 0.554), PLR (AUC = 0.552), and SII (AUC = 0.507), also failed to exhibit significant diagnostic accuracy.

**Conclusion:**

Although the APRI score was elevated in preeclamptic patients, ROC analysis indicated poor discriminatory performance, suggesting that APRI does not provide meaningful diagnostic accuracy when used alone. Further prospective studies with larger populations are needed to explore its potential role in clinical practice.

## Introduction

Preeclampsia is a complex pregnancy-related disorder that occurs in the second half of pregnancy or the early postpartum period, leading to severe complications for both maternal and fetal health [1]. It is characterized by various clinical and laboratory findings, primarily hypertension and proteinuria [2]. Although the exact pathophysiological mechanisms of the disease have not been fully elucidated, impaired placental perfusion, vascular dysfunction, and inflammatory processes are believed to play a significant role in its development [3].

In recent years, the importance of novel biomarkers in the early diagnosis and prognostic evaluation of preeclampsia has been increasingly recognized. In this context, the Aspartate Aminotransferase to Platelet Ratio Index (APRI), which is suggested to reflect liver fibrosis and inflammation, along with various blood count-derived inflammatory markers (e.g., Neutrophil-to-Lymphocyte Ratio, Platelet-to-Lymphocyte Ratio), has been the subject of growing research [4–6]. Some studies suggest that these biomarkers may be useful in predicting disease severity and monitoring the progression of preeclampsia. To achieve broader clinical acceptance, these parameters must be thoroughly investigated across different patient populations and varying degrees of disease severity [7]. In particular, the role of inflammation and liver injury markers in the course of preeclampsia remains an area requiring further research.

The APRI score is calculated by dividing the Aspartate Aminotransferase (AST) level by the platelet count and is widely used in the assessment of hepatic dysfunction [8]. Recognized as a reliable non-invasive indicator of liver fibrosis, this index has emerged as a potential biomarker not only for chronic liver diseases but also for various conditions where systemic inflammation plays a role [9]. Elevated APRI scores are associated with both hepatocyte injury and platelet consumption, suggesting that this parameter may reflect not only liver impairment but also systemic circulation and inflammatory processes.

The primary aim of this study was to compare the APRI score and blood-count-derived inflammatory markers between pregnant women with preeclampsia, gestational hypertension, and healthy controls. A secondary objective was to evaluate the diagnostic performance of APRI and selected inflammatory markers for identifying preeclampsia. We hypothesized that APRI and blood-count-derived inflammatory indices would be significantly elevated in preeclamptic patients compared to controls; however, we also explored whether these differences translate into clinically meaningful diagnostic accuracy.

## Materials and methods

This study is a retrospective case-control study conducted at Kütahya Health Sciences University, Evliya Çelebi Training and Research Hospital. The aim of the study was to evaluate the APRI score in patients with preeclampsia. Ethical approval was obtained from the Non-Interventional Clinical Research Ethics Committee of Kütahya Health Sciences University (approval date: February 13, 2024). Due to the retrospective design of the study, the requirement for informed consent was waived by the ethics committee.

### Study group and sample selection

In this study, retrospective file scans were performed on pregnant women who applied to the Obstetrics and Gynecology Clinic of Kütahya Health Sciences University, Evliya Çelebi Training and Research Hospital, between January 1, 2020, and December 31, 2023. A total of 204 pregnant women were included in the study and classified into three groups: 85 healthy pregnant women, 34 pregnant women diagnosed with gestational hypertension (HT), and 85 pregnant women diagnosed with preeclampsia. The control group consisted of healthy pregnant women who attended routine antenatal follow-up at the same institution and during the same study period. Control subjects were selected retrospectively from hospital records using a consecutive sampling approach. They had no diagnosis of hypertensive disorders of pregnancy or other exclusion criteria. No formal matching was performed; however, baseline demographic and obstetric characteristics were compared between groups to assess potential differences.

### Inclusion criteria

The study included pregnant women whose age, gravidity, parity, body mass index (BMI), and systolic and diastolic blood pressure values were available in the medical records. Additionally, gestational age at delivery, mode of delivery, neonatal birth weight, and 1- and 5-minute APGAR scores were recorded and evaluated.

### Exclusion criteria

Exclusion criteria included multiple pregnancies, pre-existing chronic diseases such as diabetes mellitus, chronic hypertension, and renal diseases, the presence of fetal anomalies, and incomplete medical records.

### Data collection

The demographic and clinical characteristics of the patients included in the study were obtained from the hospital record system. All data were retrospectively reviewed from patient files, and the following parameters were evaluated and compared among the three groups. Demographic parameters included age, gravidity, parity, and body mass index. Neonatal parameters included birth weight, neonatal gender, 1-minute and 5-minute APGAR scores, and gestational age at delivery. Blood pressure parameters involved systolic blood pressure, diastolic blood pressure, and mean arterial pressure, which was calculated as diastolic pressure plus one-third of the difference between systolic and diastolic pressure. Hematological parameters included hemoglobin, hematocrit, white blood cell count, neutrophil count, lymphocyte count, monocyte count, and platelet count. Inflammatory markers such as neutrophil-to-lymphocyte ratio (NLR), monocyte-to-lymphocyte ratio (MLR), platelet-to-lymphocyte ratio (PLR), and systemic immune-inflammation index (SII) were assessed, with the latter being calculated as platelet count multiplied by the neutrophil-to-lymphocyte ratio. Biochemical parameters included aspartate aminotransferase, alanine aminotransferase, blood urea nitrogen, and creatinine levels. All hematological and biochemical parameters analyzed in this study were obtained from blood samples collected at the same time point as APRI calculation: at the time of initial diagnosis of preeclampsia (prior to treatment and delivery) for the preeclampsia group, and during routine antenatal evaluation at hospital admission for the gestational hypertension and control groups.

Blood count–derived inflammatory indices were calculated using standard formulas. NLR was calculated as the neutrophil count divided by the lymphocyte count. MLR was calculated as the monocyte count divided by the lymphocyte count, and PLR was calculated as the platelet count divided by the lymphocyte count. The SII was calculated as platelet count × neutrophil count / lymphocyte count.

### APRI score calculation

The APRI score was calculated using laboratory parameters obtained at the time of initial diagnosis of preeclampsia, before delivery and prior to the initiation of any disease-specific treatment. For the control and gestational hypertension groups, APRI was calculated using laboratory values obtained during routine antenatal evaluation at hospital admission. The upper limit of normal for AST was accepted as 40 U/L in accordance with standard laboratory reference values [10]. Platelet counts were measured using automated hematology analyzers and reported as ×10⁹/L. The APRI score was calculated according to the following formula: APRI = [(AST / upper limit of normal) × 100] / platelet count (10⁹/L) [11]. Higher APRI values were considered to reflect an increased risk of hepatic involvement. The calculated APRI scores were statistically analyzed and compared among the three study groups to evaluate their potential association with preeclampsia severity.

### Diagnostic criteria for preeclampsia

Preeclampsia was defined according to the American College of Obstetricians and Gynecologists (ACOG) Practice Bulletin No. 222 criteria [12] as new-onset hypertension occurring after 20 weeks of gestation in a previously normotensive woman, characterized by systolic blood pressure ≥ 140 mmHg and/or diastolic blood pressure ≥ 90 mmHg measured on at least two occasions at least 4 h apart. In the presence of hypertension, preeclampsia was diagnosed when proteinuria was detected, defined as ≥ 300 mg protein in a 24-hour urine collection, a spot urine protein-to-creatinine ratio ≥ 0.3, or a dipstick reading of ≥ 2+, in accordance with ACOG criteria. Twenty-four-hour urine protein measurement was performed when clinically feasible; however, in patients with suspected severe preeclampsia or those requiring urgent delivery, rapid and clinically appropriate methods were preferred to avoid delaying management. In the absence of proteinuria, preeclampsia was diagnosed if one or more severe features were present, including thrombocytopenia (platelet count < 100,000/µL), impaired liver function (serum transaminase levels ≥ 2 times the upper limit of normal), renal insufficiency (serum creatinine > 1.1 mg/dL), pulmonary edema, or new-onset cerebral or visual symptoms. Gestational hypertension was defined as hypertension without proteinuria or severe features after 20 weeks of gestation, based on the same blood pressure thresholds.

### Statistical analysis

Statistical analyses were performed using IBM SPSS Statistics version 27.0 (IBM Corp., Armonk, NY, USA). Continuous variables were summarized as mean±standard deviation (Mean ± SD), while categorical variables were presented as frequencies and percentages. The normality of data distribution was assessed using the Kolmogorov-Smirnov test. For non-normally distributed variables, Kruskal-Wallis tests were used for comparisons among the three independent study groups. When the Kruskal-Wallis test revealed a statistically significant overall group difference, post-hoc pairwise comparisons were performed using Dunn’s test with Bonferroni correction. In the presence of three independent study groups, one-way ANOVA was used for overall group comparisons for normally distributed variables. Independent sample t-tests and Mann–Whitney U tests were explicitly used as post-hoc tests for pairwise comparisons between two groups, when an overall significant group difference was identified. Categorical variables were compared using the chi-square test. To evaluate the diagnostic discriminatory performance of NLR, MLR, PLR, SII, and APRI scores in the diagnosis of preeclampsia, receiver operating characteristic (ROC) curve analysis was performed. The area under the curve (AUC) was calculated to assess the diagnostic accuracy of each parameter. All statistical analyses were performed at a significance level of *p* < 0.05.

## Results

A total of 204 pregnant women were included in the final analysis, consisting of 85 healthy controls, 34 women with gestational hypertension, and 85 women diagnosed with preeclampsia. The mean age of the participants in the study was calculated as 30.0 ± 5.9 years. The mean number of pregnancies (gravida) was 2.4 ± 1.6, while the mean number of deliveries (parity) was 1.0 ± 1.2. The mean body mass index (BMI) of the participants was 30.9 ± 6.1 kg/m². Regarding blood pressure parameters, the mean systolic blood pressure was 137.3 ± 29.2 mmHg, the mean diastolic blood pressure was 85.7 ± 17.9 mmHg, and the mean arterial pressure (MAP) was calculated as 102.9 ± 21.0 mmHg. The mean birth weight of newborns was 2718.2 ± 760.0 g, with 51.5% of them being male. When evaluating APGAR scores, the mean APGAR score at the 1st minute was 7.8 ± 1.9, while at the 5th minute, it was 9.0 ± 1.8. The mean gestational age at delivery of the participants was 38.4 ± 1.1 weeks (Table 1).

**Table 1:** Demographic and perinatal characteristics of the participants

	Mean ± S.D.
Age (years)	30.0 ± 5.9
Gravida	2.4 ± 1.6
Parity	1.0 ± 1.2
BMI (kg/m²)	30.9 ± 6.1
Systolic Blood Pressure (mm/Hg)	137.3 ± 29.2
Diastolic Blood Pressure (mm/Hg)	85.7 ± 17.9
Mean Arterial Pressure (mm/Hg)	102.9 ± 21.0
Birth Weight (grams)	2718.2 ± 760.0
Gender (Male)	105 (51.5%)
APGAR Score at 1 min	7.8 ± 1.9
APGAR Score at 5 min	9.0 ± 1.8
Gestational Age at Delivery (weeks)	38.4 ± 1.1

Data are presented as mean ± standard deviation or number (percentage)

Abbreviations used in the table include *BMI* Body Mass Index, *APGAR* Appearance, Pulse, Grimace, Activity, and Respiration score

In the hematological assessment, the mean hemoglobin level was 12.2 ± 1.4 g/dL, the hematocrit level was 37.1 ± 3.9%, and the WBC count was 11.0 ± 3.1 (10³/µL). The neutrophil, lymphocyte, and monocyte levels were recorded as 8.26 ± 2.89 (10³/µL), 2.1 ± 0.7 (10³/µL), and 0.56 ± 0.27 (10³/µL), respectively. The mean platelet count was determined as 223.7 ± 71.7 (10³/µL). Regarding biochemical parameters, the AST level was 27.0 ± 43.3 U/L, the ALT level was 17.9 ± 38.4 U/L, the BUN level was 9.3 ± 3.4 mg/dL, and the creatinine level was 0.58 ± 0.19 mg/dL. Among inflammatory markers, the NLR was calculated as 4.68 ± 3.25, the MLR as 0.29 ± 0.17, the PLR as 126.76 ± 82.8, and the SII as 2.55 ± 2.4 (Table 2).

**Table 2:** Hematological, biochemical, and inflammatory characteristics of the participants

	Mean ± S.D.
Hemoglobin (g/dL)	12.2 ± 1.4
Hematocrit (%)	37.1 ± 3.9
WBC (10³/µL)	11.0 ± 3.1
Neutrophil (10³/µL)	8.26 ± 2.89
Lymphocyte (10³/µL)	2.1 ± 0.7
Monocyte (10³/µL)	0.56 ± 0.27
Platelet (10³/µL)	223.7 ± 71.7
AST (U/L)	27.0 ± 43.3
ALT (U/L)	17.9 ± 38.4
BUN (mg/dL)	9.3 ± 3.4
Creatinine (mg/dL)	0.58 ± 0.19
NLR	4.68 ± 3.25
MLR	0.29 ± 0.17
PLR	126.76 ± 82.8
SII	2.55 ± 2.4

Data are presented as mean ± standard deviation

Abbreviations used in the table include *WBC* White Blood Cell count, *AST* Aspartate Aminotransferase, *ALT* Alanine Aminotransferase, *BUN* Blood Urea Nitrogen, *NLR* Neutrophil-to-Lymphocyte Ratio, *MLR* Monocyte-to-Lymphocyte Ratio, *PLR* Platelet-to-Lymphocyte Ratio, *SII* Systemic Immune-Inflammation Index

There was no significant difference in age among the groups (*p* = 0.380). However, the number of pregnancies (gravida) and deliveries (parity) were lower in the preeclampsia group and found to be statistically significant (*p* = 0.033 and *p* = 0.021). The BMI in the preeclampsia group was 31.5 ± 6.5 kg/m², showing a significant difference compared to the other groups (*p* < 0.001). Regarding blood pressure values, systolic (163.8 ± 19.8 mmHg) and diastolic blood pressure (100.1 ± 14.4 mmHg) were significantly higher in the preeclampsia group (*p* < 0.001). In terms of perinatal parameters, birth weight was lowest in the preeclampsia group (2303.0 ± 801.3 g), and this difference was statistically significant (*p* < 0.001). Similarly, APGAR scores at the 1st minute (6.8 ± 2.4) and 5th minute (8.3 ± 2.4) were significantly lower in the preeclampsia group (*p* < 0.001). Gestational age was also significantly lower in the preeclampsia group, indicating earlier deliveries (*p* < 0.001). No significant difference was observed between the groups in terms of gender distribution and age (*p* = 0.480) (Table 3).

**Table 3:** Comparison of demographic, clinical, and perinatal characteristics among study groups

	Control (*n* = 85)	Gestational HT (*n* = 34)	Preeclampsia (*n* = 85)	*p* value
	Mean.±S.D./Count	Mean.±S.D./Count	Mean.±S.D./Count	
**Age (years)**	29.1 ± 5.2	30.9 ± 6.3	30.6 ± 6.4	0.380 ^b^
**Gravida**	2.6 ± 1.4	2.9 ± 2.3	2.1 ± 1.4	**0.033** ^b^
**Parity**	1.2 ± 1.1	1.0 ± 1.4	0.8 ± 1.1	**0.021** ^b^
**BMI (kg/m²)**	28.9 ± 5.5	34.1 ± 5.1	31.5 ± 6.5	**< 0.001** ^b^
**Mode of Del (Vaginal)**	33 (38.8%)	2 (5.9%)	3 (3.5%)	**< 0.001** ^b^
**Systolic BP (mm/Hg)**	110.4 ± 8.2	138.1 ± 19.8	163.8 ± 19.8	**< 0.001** ^b^
**Diastolic BP (mm/Hg)**	70.8 ± 9.0	86.8 ± 12.0	100.1 ± 14.4	**< 0.001** ^b^
**MAP (mm/Hg)**	83.9 ± 7.7	103.8 ± 13.3	121.4 ± 14.8	**< 0.001** ^b^
**Birth Weight (grams)**	3168.3 ± 483.2	2630.6 ± 592.0	2303.0 ± 801.3	**< 0.001** ^b^
**Gender (Male)**	45 (52.9%)	20 (58.8%)	40 (47.1%)	0.480 ^b^
**APGAR 1st Min**	8.6 ± 0.9	8.1 ± 1.1	6.8 ± 2.4	**< 0.001** ^b^
**APGAR 5th Min**	9.7 ± 0.9	9.2 ± 1.2	8.3 ± 2.4	**< 0.001** ^b^
**Gest Week at Birth**	38.7 ± 1.5	36.6 ± 1.8	35.8 ± 32.7	**< 0.001** ^b^

Data are presented as mean ± standard deviation or number (percentage)

Abbreviations used in the table include *BMI* Body Mass Index, *BP* Blood Pressure, *MAP* Mean Arterial Pressure, *APGAR* Appearance, Pulse, Grimace, Activity, and Respiration score

Statistically significant *p*-values are indicated in bold

Superscript letters next to *p*-values denote statistical tests used: ^a^ represents the One-Way ANOVA test, while ^b^ corresponds to the Kruskal-Wallis test

There was no significant difference among the groups in terms of hemoglobin and hematocrit levels (*p* = 0.079). The WBC count was higher in the preeclampsia group (11.7 ± 3.5 10³/µL), and this difference was statistically significant (*p* = 0.037). Monocyte levels also varied among the groups, with higher levels observed in the preeclampsia group (*p* = 0.048). Regarding liver and kidney function tests, AST levels were highest in the preeclampsia group, and this difference was statistically significant (*p* < 0.001). Similarly, BUN and creatinine levels were significantly elevated in the preeclampsia group (*p* < 0.001). No significant differences were found among the groups in terms of neutrophil, lymphocyte, ALT, and platelet levels (*p* > 0.05) (Table 4).

**Table 4:** Comparison of hematological and biochemical parameters among study groups

	Control (*n* = 85)	Gestational HT (*n* = 34)	Preeclampsia (*n* = 85)	
	Mean.±S.D.	Mean.±S.D.	Mean.±S.D.	*p* value
**Hemoglobin (g/dL)**	12.0 ± 1.5	12.1 ± 1.1	12.5 ± 1.5	0.079 ^a^
**Hematocrit (%)**	36.6 ± 4.1	36.6 ± 3.0	37.7 ± 3.8	0.138 ^a^
**WBC (10³/µL)**	10.4 ± 2.5	10.6 ± 2.9	11.7 ± 3.5	**0.037** ^**b**^
**Neutrophil (10³/µL)**	7.80 ± 2.2	8.00 ± 2.9	8.83 ± 3.4	0.100 ^b^
**Lymphocyte (10³/µL)**	1.9 ± 0.6	2.0 ± 0.6	2.2 ± 0.9	0.062 ^b^
**Monocyte (10³/µL)**	0.55 ± 0.3	0.56 ± 0.2	0.57 ± 0.2	**0.048** ^**b**^
**Platelet (10³/µL)**	226.0 ± 64.4	209.9 ± 66.9	226.8 ± 80.2	0.474 ^a^
**AST (U/L)**	20.2 ± 11.9	25.7 ± 39.8	34.3 ± 60.6	**< 0.001** ^**b**^
**ALT (U/L)**	13.1 ± 6.5	18.2 ± 41.3	22.6 ± 53.0	0.103 ^b^
**BUN (mg/dL)**	7.7 ± 1.8	9.8 ± 4.2	10.8 ± 3.7	**< 0.001** ^**b**^
**Creatinine (mg/dL)**	0.44 ± 0.09	0.64 ± 0.16	0.69 ± 0.19	**< 0.001** ^**b**^

Data are presented as mean ± standard deviation

Abbreviations used in the table include *WBC* White Blood Cell count, *AST* Aspartate Aminotransferase, *ALT* Alanine Aminotransferase, *BUN* Blood Urea Nitrogen

Statistically significant *p*-values are indicated in bold

Superscript letters next to *p*-values denote statistical tests used: ^a^ represents the One-Way ANOVA test, while ^b^ corresponds to the Kruskal-Wallis test

There was no statistically significant difference among the groups in terms of NLR, MLR, PLR, and SII (*p* > 0.05). The APRI score was highest in the preeclampsia group (0.29 ± 0.95), and this difference was statistically significant (*p* = 0.004). No significant differences were observed among the groups for other inflammatory markers (Table 5).

**Table 5:** Comparison of inflammatory markers and APRI score among study groups

	Control (*n* = 85)	Gestational HT (*n* = 34)	Preeclampsia (*n* = 85)	*p* value
	Mean.±S.D.	Mean.±S.D.	Mean.±S.D.	
**NLR**	4.37 ± 1.7	4.39 ± 2.1	5.12 ± 4.5	0.106 ^b^
**MLR**	0.30 ± 0.2	0.30 ± 0.13	0.29 ± 0.2	0.593 ^b^
**PLR**	128.90 ± 59.1	114.97 ± 51.3	129.34 ± 109.4	0.319 ^b^
**SII**	2.37 ± 1.5	2.44 ± 1.7	2.78 ± 3.1	0.247 ^b^
**APRI Score**	0.10 ± 0.05	0.19 ± 0.51	0.29 ± 0.95	**0.004** ^b^

Data are presented as mean ± standard deviation

Abbreviations used in the table include *NLR* Neutrophil-to-Lymphocyte Ratio, *MLR* Monocyte-to-Lymphocyte Ratio, *PLR* Platelet-to-Lymphocyte Ratio, *SII* Systemic Immune-Inflammation Index, *APRI* Aspartate Aminotransferase to Platelet Ratio Index

Statistically significant *p*-values are indicated in bold

Superscript letters next to *p*-values denote statistical tests used: ^b^ corresponds to the Kruskal-Wallis test

The diagnostic discriminatory performance of the APRI score in predicting preeclampsia was found to be low (AUC = 0.437, *p* = 0.128). Similarly, other inflammatory markers, including NLR (AUC = 0.539, *p* = 0.344), MLR (AUC = 0.554, *p* = 0.186), PLR (AUC = 0.552, *p* = 0.209), and SII (AUC = 0.507, *p* = 0.874), did not demonstrate significant diagnostic discriminatory performance for preeclampsia (*p* > 0.05) (Table 6).

**Table 6:** ROC analysis of inflammatory markers and APRI score for predicting preeclampsia

				Asympt. 95% CI	
	AUC	Std. Deviation	*p* value	Lower Bound	Upper Bound
APRI Score	0.437	0.041	0.128	0.357	0.518
NLR	0.539	0.042	0.344	0.456	0.622
MLR	0.554	0.041	0.186	0.474	0.635
PLR	0.552	0.042	0.209	0.469	0.634
SII	0.507	0.042	0.874	0.425	0.588

Data are presented as the area under the curve (AUC) with standard deviation, *p*-values, and 95% confidence intervals (CI)

Abbreviations used in the table include *NLR* Neutrophil-to-Lymphocyte Ratio, *MLR* Monocyte-to-Lymphocyte Ratio, *PLR* Platelet-to-Lymphocyte Ratio, *SII* Systemic Immune-Inflammation Index, *APRI* Aspartate Aminotransferase to Platelet Ratio Index

Correlation analysis revealed that the APRI score was significantly and negatively correlated with birth weight (*r* = − 0.164, *p* = 0.019), 1-minute APGAR score (*r* = − 0.335, *p* < 0.001), and 5-minute APGAR score (*r* = − 0.210, *p* = 0.003). No significant correlation was observed between APRI score and gestational age at delivery (*r* = − 0.036, *p* = 0.611) (Table 7).

**Table 7:** Correlation between APRI score and perinatal outcomes

		APRI Score
**Birth Weight (grams)**	Pearson Correlation	-0.164
	p value	**0.019**
**APGAR 1**	Pearson Correlation	-0.335
	p value	**< 0.001**
**APGAR 5**	Pearson Correlation	-0.210
	p value	**0.003**
**Gest Week at Birth**	Pearson Correlation	-0.036
	p value	0.611

Data are presented as Pearson correlation coefficients (r) with corresponding *p* values

Gestational week refers to gestational age at delivery

*APRI* Aspartate Aminotransferase to Platelet Ratio Index, *APGAR* Appearance, Pulse, Grimace, Activity, and Respiration score

Statistically significant *p* values (*p* < 0.05) are indicated in bold

## Discussion

In this study, the higher APRI score observed in the preeclampsia group supports the potential involvement of liver function impairment and highlights the significance of systemic inflammation. However, the examined inflammatory indices (NLR, MLR, PLR, SII) and the APRI score did not demonstrate significant diagnostic discriminatory performance. This finding suggests that preeclampsia involves multifaceted pathophysiological mechanisms and that a single inflammatory marker may not be sufficient for predicting the disease. Our findings may reflect possible hepatic involvement in gestational hypertensive disorders; however, given the poor discriminatory performance observed in ROC analysis, this observation should not be interpreted as evidence of early diagnostic utility. The significantly higher APRI scores observed in hypertensive pregnancies, despite the absence of statistically significant elevations in systemic inflammatory markers such as SII, NLR, MLR, and PLR, may indicate a biological association rather than a clinically useful diagnostic pattern. Although APRI levels were higher in hypertensive pregnancies compared to inflammatory indices, the cross-sectional design of the study and the very poor discriminatory performance observed in ROC analysis do not allow conclusions regarding temporal sequence or early pathophysiological events. These findings may reflect complex interactions between hepatic involvement and systemic inflammation; however, the current data do not allow conclusions regarding their temporal relationship. Previous studies have demonstrated that higher 24-hour urine protein levels are independently associated with adverse maternal and perinatal outcomes in hypertensive pregnancies, supporting the prognostic relevance of end-organ involvement beyond diagnostic thresholds [13]. Further prospective studies are needed to investigate the temporal relationship between hepatic dysfunction and systemic inflammation in preeclampsia. Consistent with these observations, our correlation analysis demonstrated significant negative associations between APRI score and birth weight as well as APGAR scores, while no significant correlation was observed with gestational age at delivery.

Preeclampsia is a significant obstetric complication that arises in the second half of pregnancy, leading to severe maternal and fetal morbidity [14]. Its pathogenesis involves mechanisms such as vascular endothelial damage, inflammation, and coagulopathy [1]. In addition to conventional diagnostic criteria, novel inflammatory markers, including the NLR and MLR, are being investigated. The APRI score, which is calculated as the ratio of AST levels to platelet count, has gained attention as a potential marker for predicting severe complications such as HELLP syndrome due to its association with liver dysfunction [15]. In this study, liver-related markers such as APRI were found to be significantly higher in the preeclampsia group compared to healthy pregnant women. Although NLR, MLR and PLR also showed an increase, these changes were not statistically significant. APRI levels were associated with liver and renal dysfunction, but its utility as a biomarker for diagnostic discriminatory performance preeclampsia was found to be limited. These findings highlight the importance of the inflammatory component in preeclampsia, emphasizing the need for close patient monitoring and the development of appropriate treatment strategies.

Özkan et al. (2022) investigated the role of APRI, DNI, NLR, PLR, and PDW in predicting preeclampsia severity in a cohort of 792 normotensive and 1213 gestational hypertension cases. Their findings demonstrated that APRI scores were significantly elevated in the severe preeclampsia (SPE) group, whereas other parameters had limited clinical significance [4]. Similarly, our study found that APRI scores were significantly higher in the preeclampsia group compared to healthy pregnant women, suggesting its potential role in preeclampsia management.

Mannaerts et al. (2019) reported that gravida, parity, gestational age, and birth weight were significantly lower in preeclamptic patients, while mean platelet volume (MPV) was elevated before the 20th gestational week, with an optimal cutoff value of 8.15 for predicting preeclampsia [16]. By the end of pregnancy, NLR and MPV remained high, whereas PLR was lower. In our study, although not statistically significant, NLR levels showed a numerical increase in the preeclampsia group, while no significant difference was observed in PLR. Additionally, APRI scores were significantly higher in preeclamptic patients. These findings further support the association between preeclampsia and inflammatory processes and align with the results of Mannaerts et al. (2019).

Gezer et al. (221 normotensive, 209 preeclamptic cases) reported that neutrophil, platelet, NLR, and PLR levels were significantly higher in the preeclampsia group, whereas hemoglobin levels were lower [17]. The study emphasized the strong predictive role of NLR and PLR in preeclampsia, identifying critical threshold values of 3.08 and 126.8, respectively. Similarly, although not statistically significant, NLR and PLR levels showed a numerical increase in the preeclampsia group (PLR: 129.34). However, there were differences in hemoglobin levels between the two studies. Despite this, both studies highlight the importance of inflammatory parameters in predicting preeclampsia. Wang et al. (2019) retrospectively analyzed data from 367 preeclamptic and 172 healthy pregnant women, finding that neutrophil, lymphocyte, and monocyte counts, along with NLR and MLR, were significantly different in the preeclampsia group compared to controls [18]. ROC analysis demonstrated that NLR and MLR had high diagnostic accuracy for preeclampsia, with NLR > 4.182 being associated with an increased risk of preterm birth and maternal-neonatal complications. In our study, although not statistically significant, NLR and other inflammatory markers showed a numerical increase in the preeclampsia group, with a mean NLR value of 5.12.

Zheng et al. (2019) conducted a meta-analysis including seven studies and reported that NLR is an important biomarker for diagnosing preeclampsia (PE), with a sensitivity of 74% and a specificity of 64%. The diagnostic odds ratio was found to be 8.44, with an AUC value of 0.82 [19]. Similarly, our study observed a numerical increase in NLR levels in the preeclampsia group compared to healthy pregnant women, without reaching statistical significance, which is consistent with the findings of the meta-analysis. Yavuzcan et al. (2014) examined hematological parameters among women with severe preeclampsia, healthy pregnant women, and non-pregnant controls. Their study found a statistically significant difference in NLR, while no significant differences were observed in MPV and PLR [20]. Our study yielded similar findings, showing a numerical increase in NLR levels without statistical significance, while PLR and MPV did not show significant variations. Kurtoğlu et al. (2014) analyzed 203 pregnant women (73 normotensive, 107 severe preeclampsia, and 23 mild preeclampsia cases) and found that gestational age, hemoglobin levels, and fetal birth weight were lower in the preeclampsia group [21]. Additionally, they reported elevated NLR levels in preeclampsia cases but found no significant relationship between NLR and disease severity, proteinuria levels, symptoms, or onset time. These findings are consistent with the trends observed in our study, without implying statistical significance.

Şaşmaz et al. (2019) compared 40 patients diagnosed with HELLP syndrome and 124 healthy pregnant controls, reporting significant differences in urine protein levels, platelet count, ALT, AST, creatinine, D-dimer levels, and APRI scores in the HELLP group [22]. Multivariate regression analysis indicated that APRI was a stronger predictor of HELLP syndrome than AST, with ROC analysis showing sensitivity and specificity values of 82.6% and 87.6%, respectively. Similarly, Tolunay et al. (2021) found that first-trimester APRI scores were significantly higher in pregnant women who later developed HELLP syndrome, reporting sensitivity and specificity values of 88.1% and 94.6%, respectively [23]. These findings support our study’s observation of elevated APRI scores in the preeclampsia group, reinforcing its clinical relevance. Özkan et al. (2022) analyzed 1,213 hypertensive pregnant women (GHT, PE, and SPE) alongside a control group, revealing that APRI scores were significantly higher in the SPE group. Additionally, NLR was identified as the best predictor for PE, while APRI and PDW were independent predictors for SPE [4]. Ipek et al. (2024) examined 258 pregnant women and found that first-trimester APRI (APRI-1) scores were significantly lower in the superimposed preeclampsia group, whereas third-trimester APRI (APRI-2) scores were notably higher. This suggests that APRI-2 may serve as a valuable biomarker for predicting preeclampsia [24]. Our findings align with these studies, confirming the elevation of APRI scores in preeclamptic cases and highlighting its potential clinical significance.

In addition to preeclampsia and HELLP syndrome, APRI-related indices have also been evaluated in other pregnancy-specific hepatic disorders. Karagün et al. demonstrated that aminotransferase-to-platelet ratios, particularly the ALT/PLT ratio, showed high diagnostic accuracy in mild intrahepatic cholestasis of pregnancy, suggesting that platelet-adjusted liver enzyme indices may reflect hepatic dysfunction across different pregnancy-related liver diseases [25]. These findings support the concept that APRI-based parameters may have broader applicability in pregnancy-associated hepatic conditions, beyond hypertensive disorders alone.

One of the main limitations of this study is its retrospective design, as data collection relied solely on patient records. This may have affected the accuracy of the results due to missing or incorrect entries. Additionally, the single-center nature of the study limits its generalizability to populations with different geographic and demographic characteristics. Multi-center and prospective studies would provide more reliable and comprehensive findings. The lack of standardized treatment management for preeclampsia and gestational hypertension may have also contributed to variability in biochemical and hematological parameters, and the effect of treatment responses on APRI scores could not be adequately assessed. Lastly, long-term maternal and neonatal outcomes were not evaluated, leaving the prognostic value of the APRI score uncertain in the later stages of pregnancy. Future prospective studies with larger patient populations and comparative biomarker analyses could further clarify the role of APRI in the diagnosis and management of preeclampsia.

This study highlights the necessity of evaluating preeclampsia through a multidimensional approach that includes both clinical and laboratory markers. Our findings indicate that although the APRI score was significantly elevated in the preeclampsia group, ROC analysis did not demonstrate sufficient diagnostic accuracy for predicting the disease. Similarly, other inflammatory parameters (NLR, MLR, PLR, SII) showed limited diagnostic discriminatory performance in identifying preeclampsia. However, the significantly higher APRI scores observed in hypertensive pregnant patients, despite the lack of statistically significant increases in systemic inflammatory markers, suggest that hepatic dysfunction may be an early pathophysiological event in gestational hypertensive disorders. This raises the possibility that subclinical hepatic microdamage might precede the inflammatory response, which becomes more evident in advanced stages of the disease. Given the complex pathophysiology of the condition, relying on a single parameter may not adequately reflect the entire disease process. Future studies with larger patient populations and multi-center prospective designs could provide a better understanding of the clinical utility of APRI, particularly in reflecting liver function impairment and its potential role in the progression of preeclampsia. Additionally, a more comprehensive approach incorporating other biomarkers and risk factors may offer valuable insights into the diagnosis and management of preeclampsia. Importantly, these findings provide clinically relevant negative evidence by demonstrating that commonly used and easily accessible inflammatory indices should not be overinterpreted as diagnostic tools for preeclampsia. By clearly defining the limitations of APRI and related markers, this study contributes to more rational and evidence-based use of laboratory parameters in obstetric practice.

## Data Availability

The datasets generated and analyzed during the current study are available from the corresponding author upon reasonable request. Due to ethical considerations and institutional regulations, access to patient data is restricted and requires appropriate approvals.
